# Supplementary material for: A New Chromosome-Assigned Mongolian Gerbil Genome Allows Characterization of Complete Centromeres and a Fully Heterochromatic Chromosome
Source: Mol Biol Evol. 2023 May 15;40(5):msad115. doi: 10.1093/molbev/msad115 (PMC10210625; doi:10.1093/molbev/msad115)
Supplement: msad115_Supplementary_Data [file Supplemental_Material_2_Genome_sketch_HiRes_v2.pdf › Supplemental_Material_2_Genome_sketch_HiRes_v2.pdf]

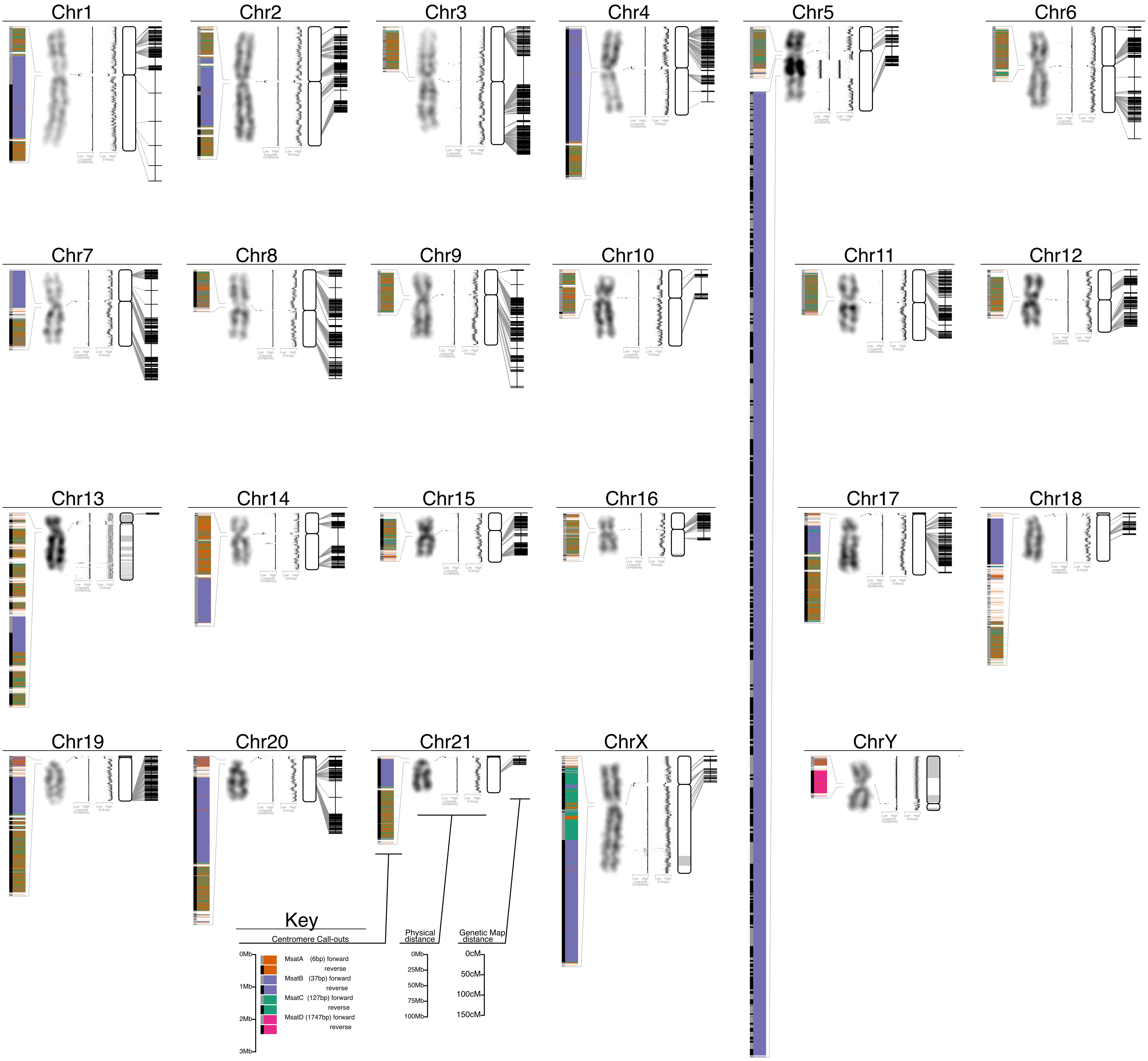
Chr1
Chr2
Chr3
Chr4
Chr5
Chr6
Chr7
Chr8
Chr9
Chr10
Chr11
Chr12
Chr13
Chr14
Chr15
Chr16
Chr17
Chr18
Chr19
Chr20
Chr21
ChrX
ChrY
Low High
Linguistic Complexity
Low High
Entropy
Key
Centromere Call-outs
0Mb
1Mb
2Mb
3Mb
MsatA (6bp) forward
reverse
MsatB (37bp) forward
reverse
MsatC (127bp) forward
reverse
MsatD (1747bp) forward
reverse
Physical distance
0Mb
25Mb
50Mb
75Mb
100Mb
Genetic Map distance
0cM
50cM
100cM
150cM
